# Rice sucrose transporter 1, *OsSUT1*, confers to alleviation of the high temperature stress in rice grain filling

**DOI:** 10.5511/plantbiotechnology.25.0706a

**Published:** 2025-12-25

**Authors:** Hiroaki Kusano, Kao-Chih She, Kana Matsubara, Lei-Lei Wang, Kasumi Tsujiuchi, Rina Matsumoto, Masaya Shiraishi, Yo Okubo, Hiroto Yasui, Tadamasa Sasaki, Hiroaki Shimada

**Affiliations:** 1 Department of Biological Science and Technology, Tokyo University of Science, Katsushika, Tokyo 125-8585, Japan

**Keywords:** grain filling, high-temperature stress, map-based cloning, QTL mapping, sucrose transporter

## Abstract

Quantitative trait loci involved in reducing high temperature injury during grain filling were investigated using fifty-four lines of the chromosome segment substitution lines (CSSLs) of the indica cultivar Kasalath in the japonica cultivar Nipponbare background. The ratio of chalky seeds was examined when they were ripened under the high-temperature conditions. Among these lines, SL13 seeds showed an obvious trait of alleviating injury. In SL13, a part of the chromosome 3 was substituted with the Kasalath genome, which contained a locus of this trait locus. Fine mapping of this locus using the progenies obtained by crossing SL13 with Nipponbare narrowed it down to a region of 3.36–3.81 Mb on chromosome 3. This region included the *OsSUT1* gene encoding sucrose transporter 1. The Nipponbare *OsSUT1* contained 19-nucleotide insertion in the promoter region, suggesting that this diversity might affect the transcription level of this gene. The progeny plants of SL13 containing the Kasalath *OsSUT1*, which had a significantly higher expression level of this gene, obviously reduced the high-temperature injury. Transformants carrying the Kasalath *OsSUT1* gene showed significant alleviation in the high-temperature injury during grain filling. Our results indicate that *OsSUT1* is a major factor consisting of the locus involved in reducing the high temperature injury in SL13. These results suggest that the enhanced function of *OsSUT1* provides sufficient carbon assimilates to immature seeds even under high temperature conditions, leading to normal seed formation.

## Introduction

Global warming and climate change caused by increased greenhouse gas emissions is a major factor in reducing grain productivity. When developing rice seeds were exposed to high temperature stress during the ripening stage, they often produce in chalky and mealy endosperm due to damaged seed ripening (Morita et al. 2004; Tashiro and Wardlaw 1991). At this time, the expression level of genes involved in the synthesis of storage starch decreases. It has also been reported that the function of genes involved in starch synthesis, such as amylase, increases (Mitsui et al. 2016; Yamakawa et al. 2007). It has been suggested that ATP synthesis is reduced in immature seeds exposed to high temperature stress during the ripening stage, which leads to insufficient accumulation of storage substances and the formation of small seeds and endosperm with a chalky or white core. Transgenic plants with enhanced ATP synthesis in immature seeds show reduced ripening damage caused by high temperature (Kusano et al. 2016; She et al. 2010b). It has also been revealed that there are factors that alleviate high temperature injury to the grain filling. To date, quantitative trait loci (QTLs) involved in high temperature tolerance in rice have been analyzed, and attempts have been made to identify the genes involved (Kobayashi et al. 2013; Li et al. 2003; Murata et al. 2014; Tabata et al. 2007; Takehara et al. 2018).

A chromosome segment substitution line (CSSL) is a progeny lines obtained by crossing two lines and backcrossing the resulting F_1_ with one of the parent lines multiple times, in which only a portioned chromosome has been substituted with the genome of one of the parents (Ebitani et al. 2005; Kato and Hirayama 2021; Yasui et al. 2010). In each CSSL, the region of the genome where the genome has been substituted has been identified by the genomic analysis using the molecular markers. A CSSL covering the entire genome regions is established. These lines are accumulated and organized (Nagata et al. 2023).

When each of CSSLs is cultivated under the same growth condition and evaluated for a specific trait, many CSSLs show the traits similar to that of the major parent line of the genetic background. If a significant difference in trait is observed between the CSSL and the parent line, it is suggested that this trait originated in the gene in the substituted genomic region. Therefore, CSSLs can be used as a powerful research tool for quantitative trait analysis (Kubo et al. 2002; Suralta et al. 2008). To date, rice CSSLs have been used to identify QTLs involved in heading date (Ebitani et al. 2005), QTLs for culm length, plant height, panicle number, chlorophyll content, and leaf weight (Kanbe et al. 2008), and QTLs for lowering cadmium accumulation in rice grain (Sato et al. 2011). In relation to high temperature stress, the CSSL-based studies have identified *Apq1*, which is associated with heat resistance during grain filling (Murata et al. 2014), *qPGC11*, which mitigates grain chalkiness caused by high temperature injury (Yang et al. 2021), and *qPSLht4.1*, which confers high temperature tolerance at the flowering stage (Zhao et al. 2016).

In this study, we mapped the gene involved in tolerence to high temperature stress using a CSSL in which a part of the Nipponbare genome was substituted with the Kasalath genome. As a candidate gene, we found the *OsSUT1* gene, which contributed to reducing damage by high temperature stress during the grain filling stage.

## Materials and methods

### Rice cultivars, mutants, and growth condition

*Oryza sativa* *japonica* cv. Nipponbare, as a wild type and the host of transgenic plants, was grown in a greenhouse. For the QTL mapping, we used 54 lines of the CSSLs of the *indica* cultivar Kasalath in the *japonica* cultivar Nipponbare background (Nipponbare/Kasalath CSSLs), which are available at the National Agriculture and Food Research Organization (NARO), Tsukuba, Japan. The mapping population carries a few chromosome segments of Kasalath (*indica*) in each line overlapping with segments on a Nipponbare (*japonica*) genetic background (Supplementary Figure S1).

### Observation to grain chalkiness phenotype

To determine the damage caused by high temperature stress during seed developent, tillers of the test line were divided into two plants at mid-vegetative growth stage to generate two individuals with the same genetic background. After these transformants headed, one was subjected to high temperature ripening in a high temperature environment from the 5th to the 15th day after flowering, while the other was subjected to ripening in a normal environmental condition. The characteristics of the seeds produced by these plants was analyzed as the seeds that were developed under the normal and high temperature conditions.

For the high-temperature condition, we subjected the plants to high-temperature stress by transferring the plants into a growth chamber with settings of 33°C 12-h day and 28°C 12-h night temperature for 10 days from the 5th day after flowering as described previously (She et al. 2010b). In the control experiment with the normal temperature condition, the plants were grown in a growth chamber throughout the seed development with a 12 h/12 h day/night cycle at 28°C/25°C. The mature seeds were harvested at around 40 days after flowering. White core and other phenotypes of the endosperm were determined by illumination using backlight as described previously (Kusano et al. 2016; She et al. 2010b). The proportion of grains with chalkiness phenotypes was determined using randomly selected 20–50 grains. CSSL lines and their progenies, which showed more than 50% of normal grains, were identified the lines with alleviation of high temperature damage.

### Genome mapping of the locus for the factor involved in tolerance to high temperature stress during seed developent

Fine mapping of the locus involved in tolerance to high temperature stress was performed according to the previously described procedure (She et al. 2010a). A set of CSSLs in which a part of the Nipponbare genome was replaced with the Kasalath genome was used for genome mapping. The candidate CSSL was crossed to Nipponbare to obtain the progenies (B_1_ lines), followed by selfed pollination to produce successive progenies (B_2_, B_3_ and B_4_). B_2_, B_3_ and B_4_ lines were subjested to observation of grain appearance quality. Genotypes of these lines were determined by analysis using the established DNA SSR markers in the chromosome 3 (https://www.scribd.com/doc/148187988/SSR-Marker-List (Accessed Dec 8, 2025)). Primer sets for the molecular markers used for this analysis were listed in Supplementary Table S1.

### Analysis of the *OsSUT1* gene and its transcript

Genomic DNA was extracted according to the method of Murray and Thompson (1980). PCR was performed using Blend-taq thermo-stable DNA polymerase (Toyobo, Osaka, Japan). Total RNA was extracted from each tissue as described previously (Imamura et al. 2007). The first-strand cDNA was synthesized from 1 mg of total RNA using a ReverTra-Ace cDNA synthesis kit (Toyobo) with an oligo-dT(20) primer. Real-time quantitative PCR was performed using a QuantStudio 3 (Thermo Fisher Scientific, Waltham, MA, USA) with an SYBR Green Real-time PCR mix (Toyobo). The expression level of *OsSUT1* was determined using a primer set: 5′-cttccctcaggtggtcatcg-3′ and 5′-cgatgaccacctgagggaag-3′. The value of *Actin I* mRNA (accession number AK100267) was used for data normalization as a positive control using a primer set, 5′-ccctcctgaaaggaagtacagtgt-3′ and 5′-gtccgaagaattagaagcatttcc-3′.

### Generation of a transgenic rice plant containing a gene for expressing *OsSUT1*

The 8.4 kb fragment for the entire *OsSUT1* gene (*Os03g0170900*) including the promoter region was amplified from the genomic Kasalath DNA. The amplified fragment was inserted into pGWD1 (Nakagawa et al. 2007) to generate a binary vector for rice transformation. The resultant plasmid was used for transformation of rice cells by the Agrobacterium-mediated rice transformation procedure (Hiei et al. 1994). The regenerated rice plants were grown in the greenhouse.

## Results

### Detection of the genes related to mitigating high temperature damage to rice ripening using Nipponbare/Kasalath CSSLs

**Figure 1:**
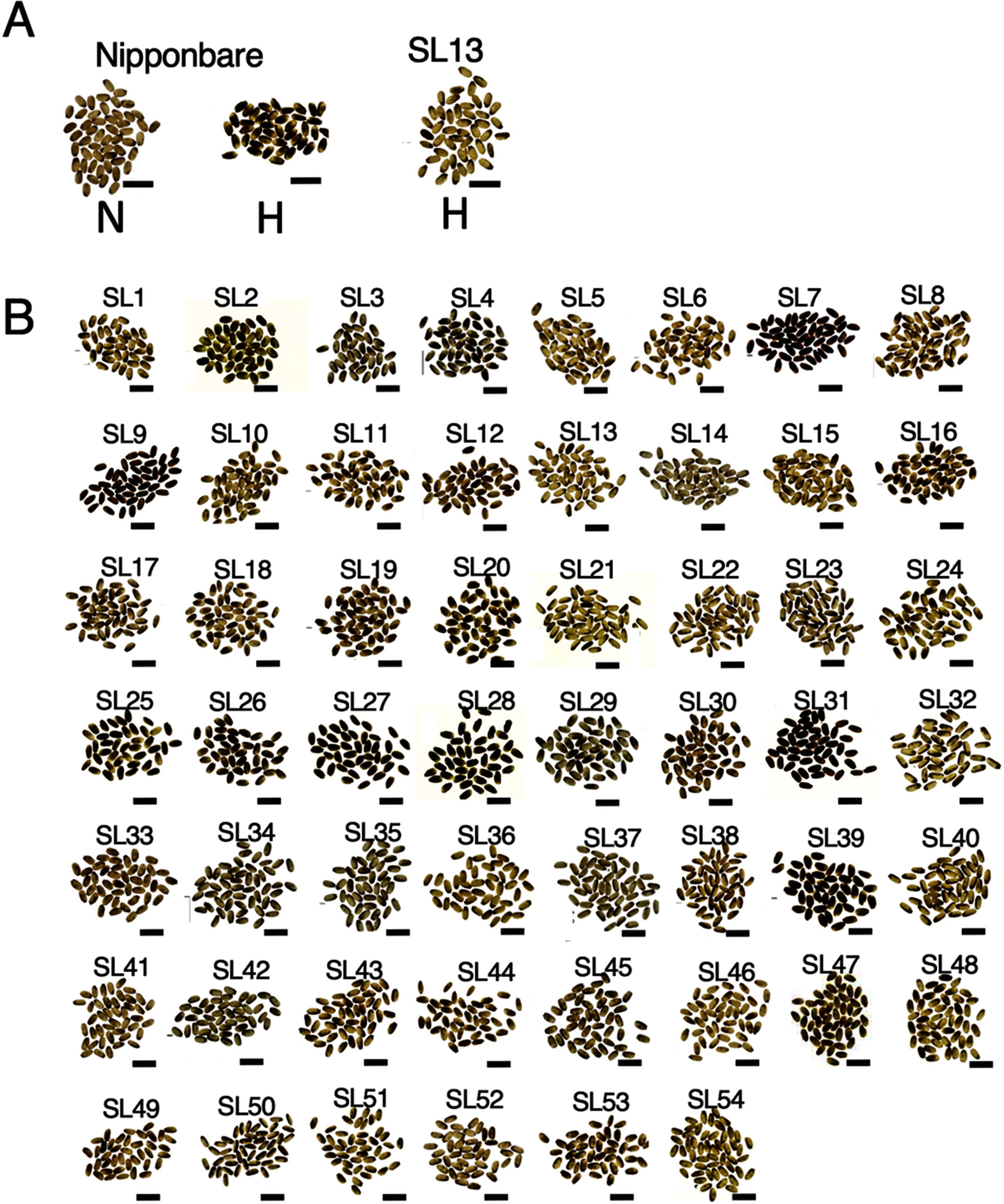
Figure 1. Appearance of mature grains of CSSLs. (A) Phenotypes of Nipponbare and SL13 grains developed in the normal (N) and high-temperature (H) environment. (B) Appearance of mature seeds of CSSLs developed in the high-temperature condition. Phenotypes of Nipponbare and CSSL grains developed in the high-temperature condition are shown. Names of CSSLs are indicated with line number with the prefix “SL”. Photographs were taken under backlight illumination. Bars, 1.0 cm.

To identify a gene involved in tolerance to high temperature stress, we examined the ripening of 54 lines of the CSSL, which contain Kasalath chromosome segments in Nipponbare background, under high temperature conditions. The parent strain Nipponbare showed the feature of chalky endosperm in its seeds, when they were ripened under the high temperature condition (Figure 1A). Large differences in degree of seed chalkiness were detected among the CSSLs. Among these CSSLs, 10 lines, SL5, SL13, SL14, SL21, SL22, SL32, SL36, SL37, SL40, SL41, SL46, and SL52, had a very low percentage of chalky seeds (Figure 1 and Supplementary Table S2). This suggests that these lines contain some factors that contributed to alleviate high temperature injury during ripening derived from Kasalath.

Among these, SL13 showed good characteristics for the alleviation of the damage by the high temperature stress during grain filling stage (Figure 1). SL13 contained the genomic fragments derived from the Kasalath genome in the chromosomes 3, 5, 7, 8, 10, and 11 (Supplementary Figure S1). The Kasalath genomic region in the region of 0 to 46.4 cM on chromosome 3 contained in SL13 was also present in SL2, SL14, SL28, SL32 and SL37, among which SL14, SL32 and SL37 showed the obvious characteristics of the alleviation of high temperature damage (Figure 1B). Other Kasalath-derived genomic regions in SL13 were contained in other CSSLs, but many of them did not show alleviation of high temperature ripening injury. These results suggest that the alleviation factors contained in SL13 are present in this region of chromosome 3.

### Identification of the factor involved in tolerance to the high temperature stress during ripening

The region of 0–46.4 cM on chromosome 3, which was substituted by the chromosome fragment from the Kasalath genome in the SL13 line, corresponds to the region of 0–10.24 Mb in the nucleotide sequence of the rice genome. SL13 was backcrossed with Nipponbare to obtain the progeny population (B_1_) and its selfed progenies (B_2_). The B_2_ lines were analyzed to narrow the region involved in the alleviation of high-temperature injury.

**Figure 2:**
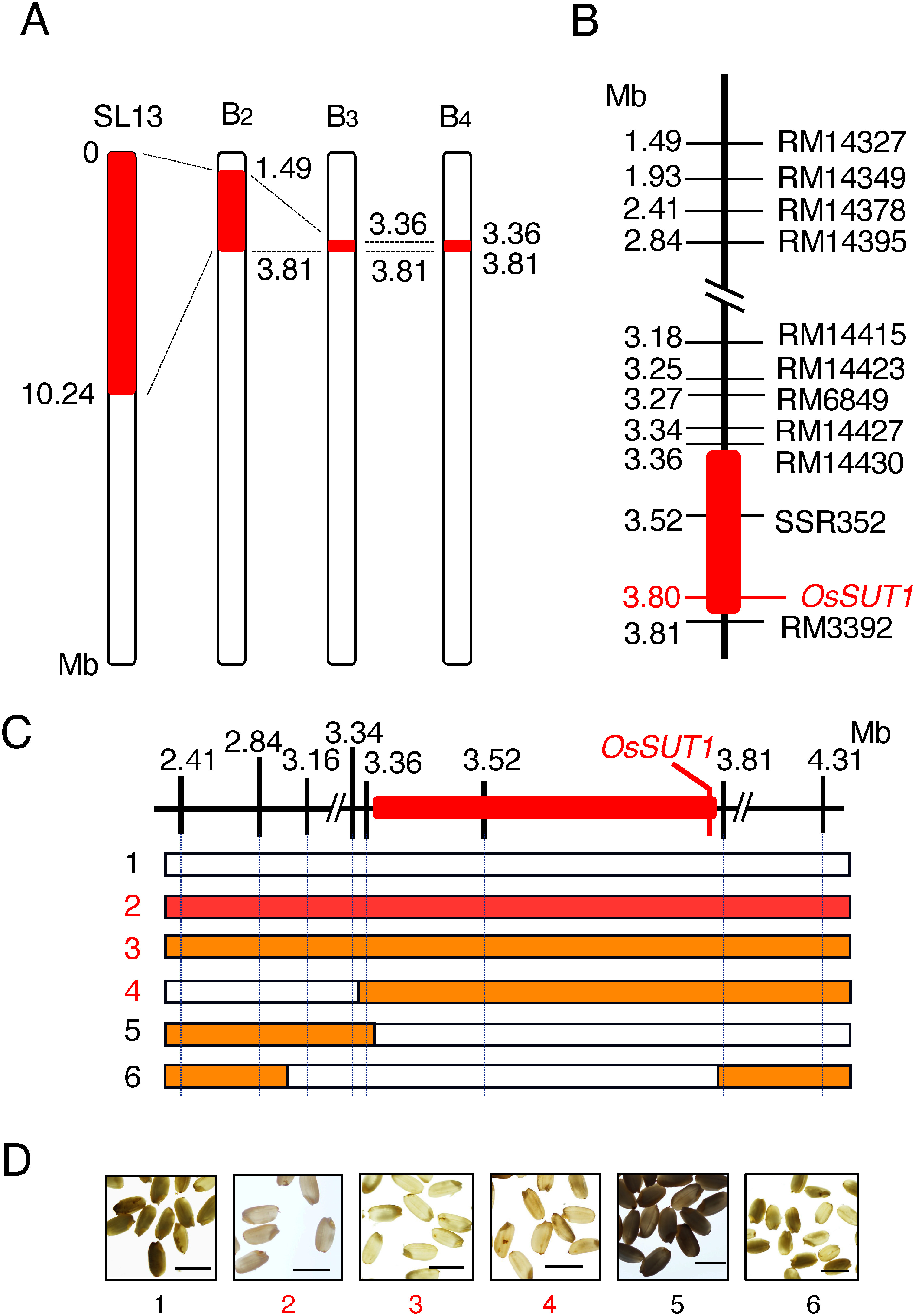
Figure 2. Mapping of the locus on the alleviation of the high-temperature injury during grain filling stage. (A) Fine mapping of the locus involved in tolerance to high-temperature stress. Regions of the Kasalath genome in chromosome 3 of SL13 and its progenies (B_2_, B_3_ and B_4_) are shown. The Kasalath genome regions are indicated by red boxes. Numbers in the figure show the position of the nucleotides in the chromosome 3. (B) Chromosome location of the region including the locus of alleviation to the high-temperature injury in B_4_. Molecular markers are shown in the right and their molecular position are indicated in the left of the bar. The red box indicates the region containing the locus involved in tolerance to high-temperature stress. (C) Substitution mapping of the reperesentative B_3_ lines (lines 1–6). Vertical bars on the upper panel indicate the physical distance (Mb) of the molecular markers tested. Position of *OsSUT1* is depicted at the position of 3.80. The region that was narrowed by the mapping is shown by the red box. The lower panels show the maps of substitution segments in the B_3_ lines (numbers 1–6). White and red blocks represent homozygous genotypes of Nipponbare and Kasalath, respectively. Orange blocks represent the hetelozygous genotype consisting of Nipponbare and Kasalath genomes. (D) Appearance of mature grains of lines 1–6 developed in the high-temperature environment. Numbers of the lines showing the phenotype of stress tolerance are indicated by red letters. Photographs were taken under backlight illumination. Bars, 1.0 cm.

Using the DNA markers located on chromosome 3, we analyzed the region of the chromosome fragment substituted with the Kasalath genome in the B_2_ plants. In parallel, we examined whether these B_2_ had the trait for the tolerance to the high-temperature stress. This analysis narrowed this locus down to the region of 1.49–3.81 Mb on chromosome 3. Next, this locus was more precisely mapped using the selfed progenies (B_3_) of these B_2_ plants. This analysis showed that this factor was located within the region of 3.36–3.81 Mb. However, further analysis using their progenies (B_4_) did not narrow the locus of this trait (Figure 2).

**Figure 3:**
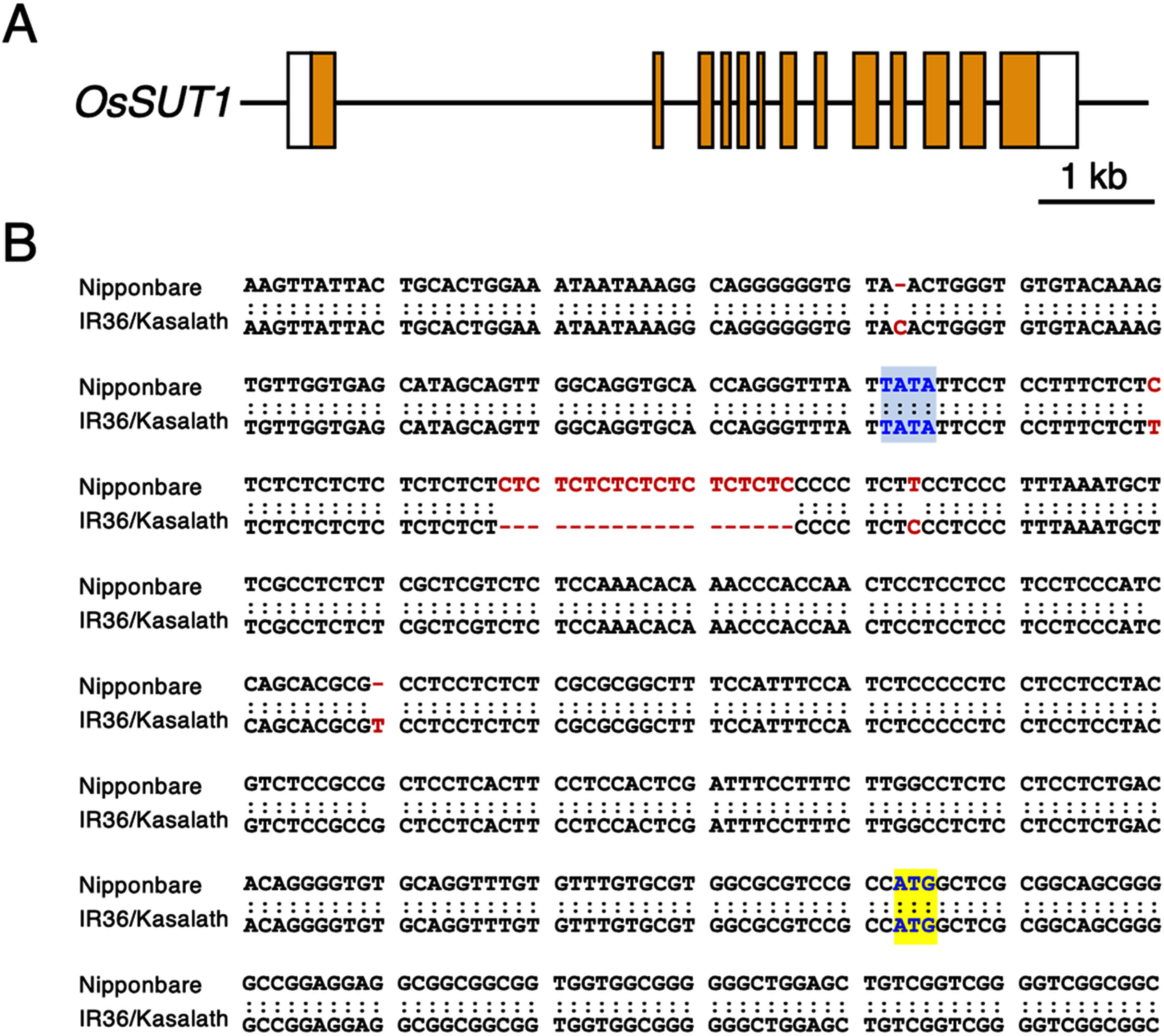
Figure 3. Structure of the *OsSUT1* gene. (A) Exon-intron structure. Open and orange boxes indicate the untranslated and coding regions in exons. (B) Nucleotide sequence of the promoter and the region around the initiation codon. Upper and lower lines show the nucleotide sequences of Nipponbare (*japonica*) and IR36/Kasalath (*indica*) *OsSUT1* genes. Polymorphic nucleotides between them are indicated by red letters. Gaps show the insertion/deletion sites. Predicted TATA box and initiation codon (ATG) are shown by blue-shaded and yellow-shaded boxes, respectively.

It was suggested that 64 genes existed in the 3.36–3.81 Mb region of chromosome 3. Of these, eight genes showed polymorphisms between the nucleotide sequences of the Nipponbare and *indica* cv. 93-11 genes, whose nucleotide sequence had been registerd (Supplementary Table S3). In these genes, *OsSUT1* (sucrose transporter1, *Os03g0170900*), which was involved in sucrose transport, was found at the location of 3.80 Mb as a candidate gene. Comparison of the nucleotide sequences of the *OsSUT1* genes from *japonica* (Nipponbare, acc. no. AP008209) and *indica* (IR36, acc. no. AF280050) revealed that a 19-nucleotides insertion was found in the promoter region located 260-bp upstream of the initiation codon of the Nipponbare gene (Figure 3). We isolated the Kasalath *OsSUT1* gene and determined that its nucleotide sequence was identical to that of the IR36 genes (Figure 3). The protein coding regions were identical and there were no polymorphisms in the *japonica* and *indica* genes.

### Analysis of *OsSUT1* expression levels in Nipponbare and Kasalath

**Figure 4:**
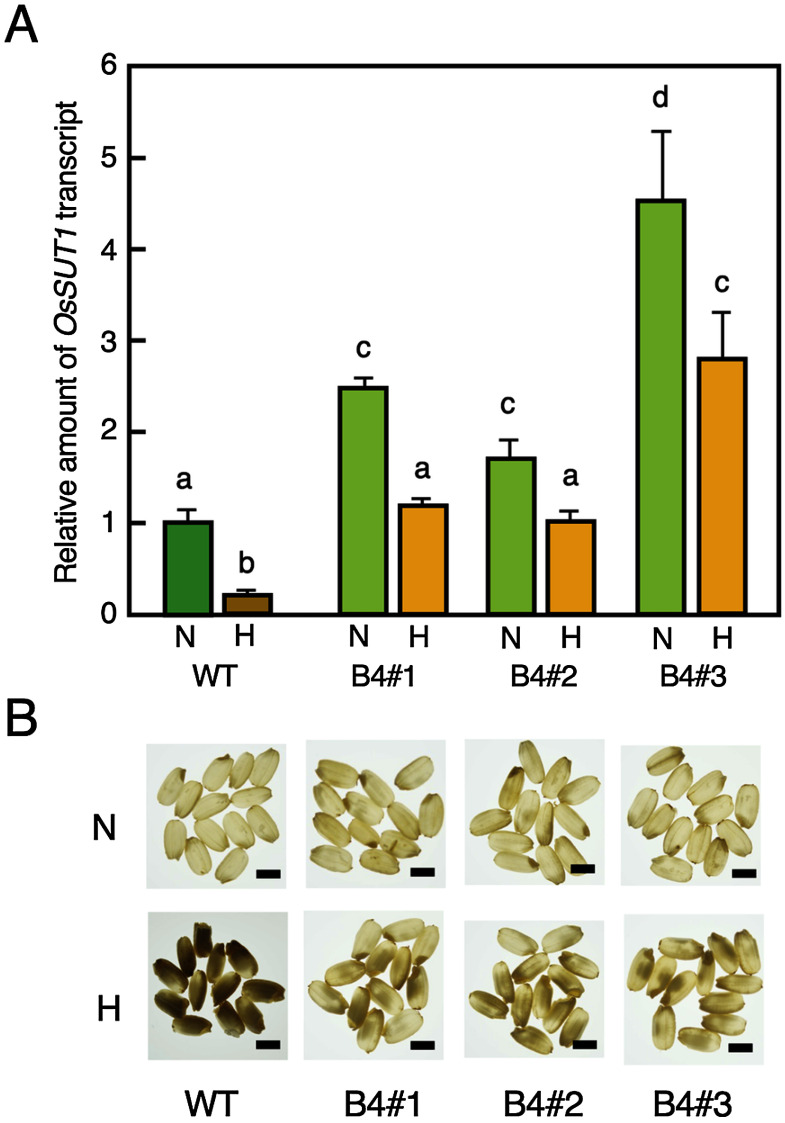
Figure 4. Observation of grain appearance quality of B_4_ progenies. (A) Expression analysis of *OsSUT1* in immature seeds of B_4_ lines (lines B4#1, B4#2, and B4#3), which contain the homozygous Kasalath alleles. Seeds were developed under the normal (N) and high (H) temperature conditions. Transcript abundance was analyzed by real-time quantitative PCR. Amounts are shown relative values to those of rice *actin1* gene. Mean values (±SD) are shown. Values are statistically analyzed by Tukey’s test (*n*=3). (B) Appearance of mature grains of WT and B_4_ lines (lines B4#1, B4#2, and B4#3) developed in the normal (N) and high-temperature (H) conditions. Photographs were taken under backlight illumination. Bars, 0.5 cm.

We analyzed the expression level of the *OsSUT1* gene in immature seeds 10 days after flowering of the B_4_ lines, B4#1, #2, and #3, in which the 3.36–3.81 Mb region of chromosome 3 was substituted with the Kasalath genome. The amount of *OsSUT1* transcript in the B_4_ lines was higher than that in Nipponbare when grain filling was performed under the normal temperature condition. When seeds were ripened under the high temperature condition, the amount of the *OsSUT1* transcript was significantly reduced in Nipponbare compared with those ripened under the normal temperature condition. However, the degree of reduction in *OsSUT1* expression in B_4_ lines was smaller than that in Nipponbare, and the value was comparable to that of Nipponbare ripened at the normal temperature (Figure 4A). Seeds of these B_4_ lines showed the properties that alleviated high-temperature injury (Figure 4B). This suggests that individuals containing the Kasalath *OsSUT1* increased its transcript, resulting in the acquisition of a trait of tolerance to high temperature stress during grain filling.

### Transformants containing the Kasalath *OsSUT1* gene

**Figure 5:**
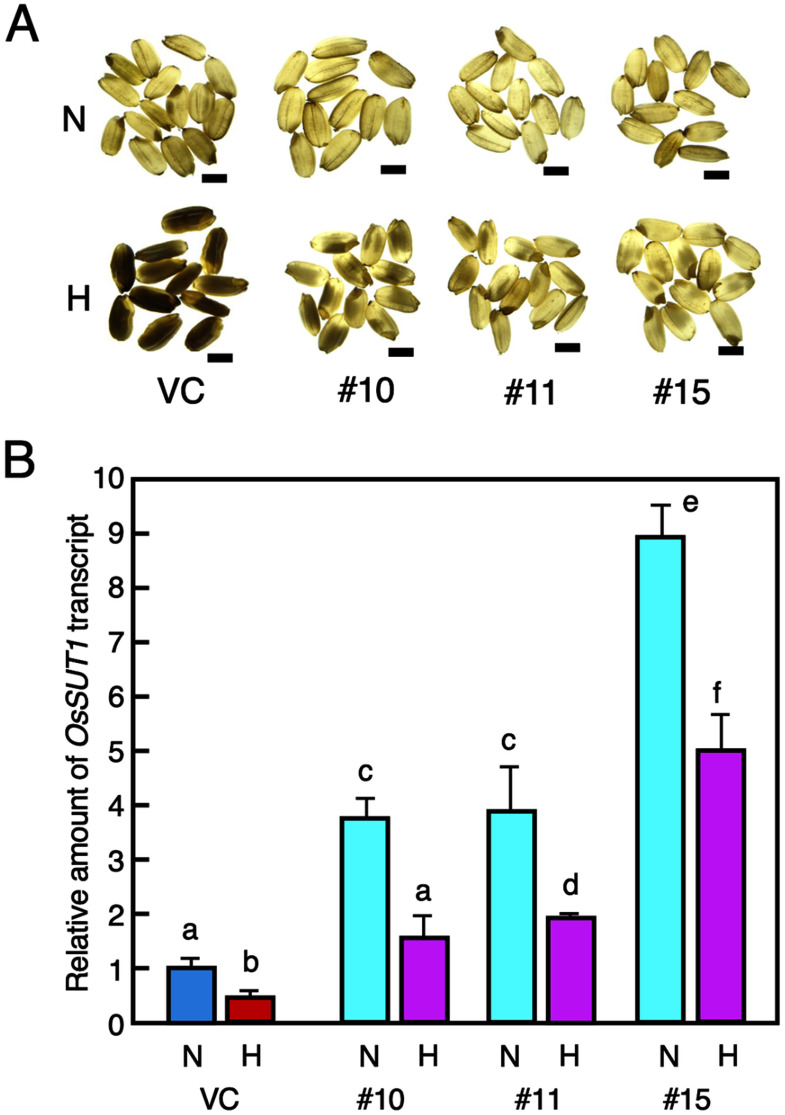
Figure 5. Transformants harboring the Kasalath *OsSUT1* gene. (A) Appearance of mature grains of vector control (VC) and transformants #10, #11, and #15 developed in the normal (N) and high-temperature (H) environment. Photographs were taken under backlight illumination. Bars, 1.0 cm. (B) Expression analysis of *OsSUT1* in immature seeds of VC and the transformants. Seeds were developed under normal (N) and high (H) temperature conditions. Transcript abundance was analyzed by real-time quantitative PCR. Amounts are shown relative values to those of rice *actin1* gene. Mean values (±SD). Values are statistically analyzed by Tukey’s test (*n*=3).

We examined whether the transformants could acquire a trait that conferred tolerance to the high-temperature stress during grain filling. To know this, the Kasalath *OsSUT1* gene was introduced into Nipponbare. Three *OsSUT1* transformants and vector control containing pGWB1 (VC) were cultivated. Their seeds were developed under the normal and high temperature conditions. High-temperature injury was observed in seeds of VC ripened under high temperature conditions, but the *OsSUT1* transformants (#10, #11, and #15) showed significant reduction in the degree of injury due to high-temperature stress (Figure 5A).

We examined the amount of the transcript of the *OsSUT1* gene in immature seeds of the *OsSUT1* transformants. When the seeds were matured under the normal temperature condition, the amount of *OsSUT1* transcripts in the *OsSUT1* transformants was significantly higher than that in VC. When they were matured under the high-temperature condition, the amount of *OsSUT1* transcripts was significantly reduced than those matured under the normal temperature condition in both the transformants and VC. In the immature seeds of the transformants, the amount of the transcript in the *OsSUT1* transformant was detected to be higher than that in VC seeds that were ripened under the normal temperature condition (Figure 5B). These results strongly indicated that these transformants showed acquisition of the sufficient tolerance to the high-temperature stress during grain filling stage.

## Discussion

There is a great need to develop new cultivars that can tolerate high temperature stress because global warming has a significant impact on the yield and quality of rice. Some traditional cultivars have a tolerance to the high temperature stress. Based on the differences in the characteristics of each cultivar, there are effective methods to reduce the damages (Morita et al. 2016; She et al. 2012). Many studies have been conducted on QTLs involved in tolerance to the injury due to the high temperature stress (Kobayashi et al. 2013; Tan et al. 2000). Recently, the *Apq1* gene has been determined as one of the QTL genes that improves the grain quality under high temperature stress (Murata et al. 2014). Identifying these factors will lead to the development of new cultivars that can be produced stably even in high temperature environments.

Using the CSSLs in which the *indica* genome was partially substituted into the Nipponbare genome background, we suggested a novel locus involved in alleviating damage caused by high temperature stress. This locus was detected on the Kasalath-derived genomic region on chromosome 3 in SL13. Fine mapping revealed that this locus located in the 3.36–3.81 Mb region on chromosome 3 (Figures 1, 2).

We compared the Nipponbare genome with the *indica* 93-11 genome in the corresponding regions because the entire nucleotide sequence was only available for the *indica* cultivar 93-11 genome. In these genomes, most genes were highly conserved although there were many differences in nucleotide sequences of the intervening region (Supplementary Table S3). In this region, seven genes contained nucleotide differences in their coding regions that were predicted to result in the additions, deletions, or substitutions of a few amino acid residues.

The *OsSUT1* gene located at 3.80 Mb contained a 19-nt insertion/deletion in the promoter region, which consisted of a simple repeat sequence with difference in number of CT repeats (Figure 3). This structural difference was found in Kasalath and IR36, suggesting to be conserved in other *indica* genes. This region may affect the expression level of the gene (Figure 3). It has been reported that high temperature stress during the grain filling stage alters the expression levels of the genes involved in sugar metabolism (Guo et al. 2008; Takehara et al. 2018; Yamakawa et al. 2007). *OsSUT1* encodes a sucrose transporter 1 and has been suggested to be involved in sugar transport in the sink organs (Wu et al. 2021).

The expression levels of *OsSUT1* were significantly higher in the B_4_ lines of the progenies of SL13 that showed the alleviation of injury due to high-temperature stress. The amount of this transcript maintained a large level in immature seeds even when they were developed under the high temperature condition (Figure 4). This result suggested that there is a correlation between the alleviation of high temperature injury to ripening and the amount of *OsSUT1* transcript.

Transformants carrying the Kasalath *OsSUT1* gene showed significant alleviation to the high temperature injury during grain filling stage (Figure 5A). The transcripts of *OsSUT1* were significantly increased in the immature seeds of these transformants (Figure 5B). This fact indicates that the Kasalath *OsSUT1* gene results in a higher transcription level than the Nipponbare gene. Individuals carrying Kasalath *OsSUT1* clearly showed a reduction in damage caused by high temperature stress during grain filling. Our results indicate that *OsSUT1* is a major factor consisting of the QTL involved in reducing the high temperature injury in SL13. It is suggested that the enhanced function of *OsSUT1* through increased expression levels provides sufficient carbon assimilates to immature seeds even under high temperature conditions, leading to the formation of seeds with normal morphology.

## Abbreviations

PCR: polymerase chain reaction

## Description of supplementary files

Supplementary Table S1. List of PCR primers used for the SSR markers.

Supplementary Table S2. Evaluation of grain appearance quality of CSSLs.

Supplementary Table S3. List of genes predicted in the 3.36–3.81 Mb region of chromosome 3.

Supplementary Figure S1. Schematic representation of the genotype data of Nipponbare/Kasalath CSSLs and the *OsSUT1* locus.
