# Supplementary material for: Rice sucrose transporter 1, OsSUT1, confers to alleviation of the high temperature stress in rice grain filling
Source: Plant Biotechnol (Tokyo). 2025 Dec 25;42(4):467–73. doi: 10.5511/plantbiotechnology.25.0706a (PMC12781913; doi:10.5511/plantbiotechnology.25.0706a)
Supplement: Supplementary Data [file plantbiotechnology-42-4-25.0706a-s001.pdf]

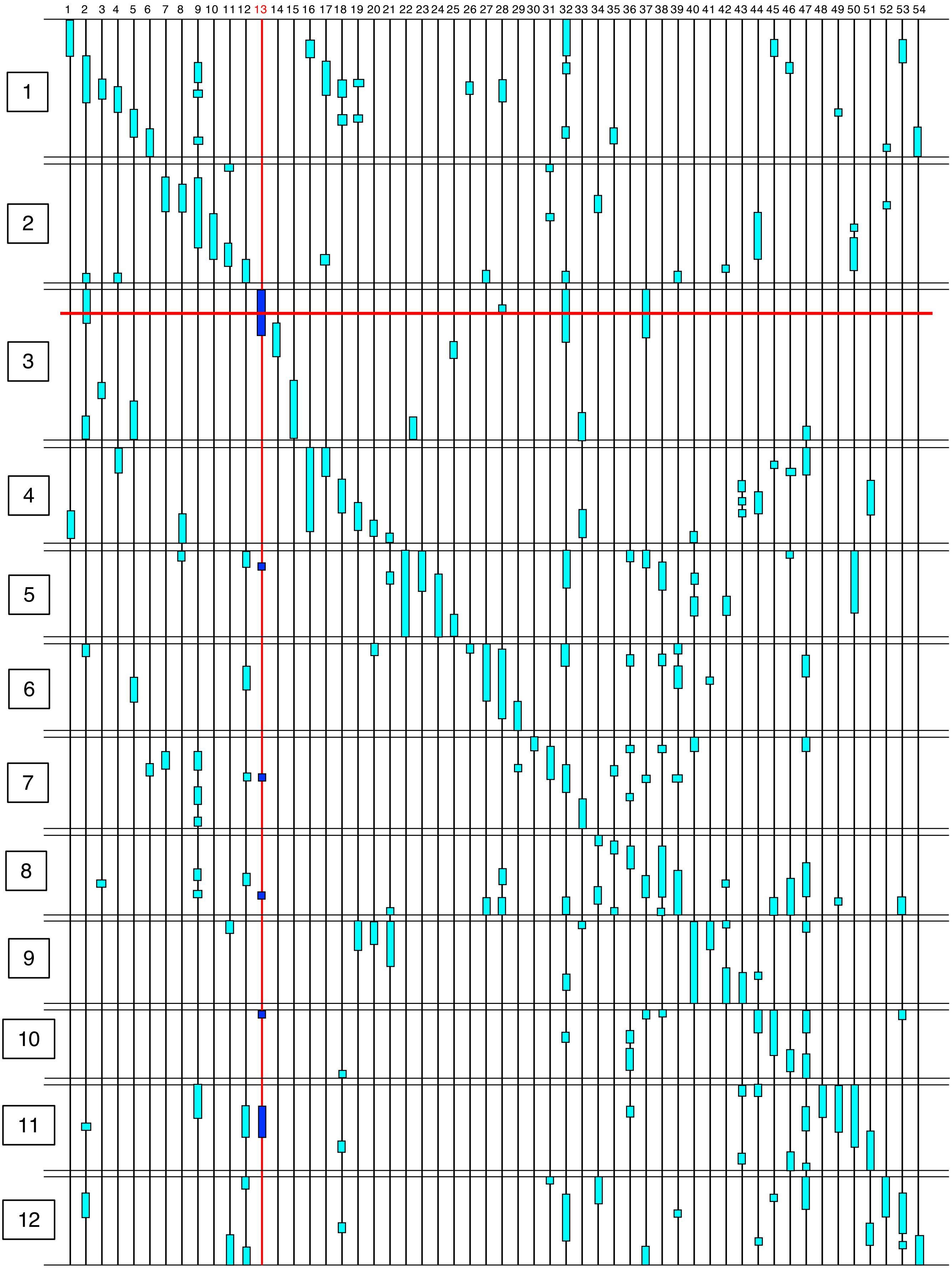

Supplementary Figure S1. Schematic representation of the genotype data of Nipponbare/Kasalath CSSLs and the *OsSUT1* locus. List of 54 lines of the CSSLs of the *indica* cultivar Kasalath in the *japonica* cultivar Nipponbare background are indicated. The horizontal columns indicate the names of CSSL shown without the prefix "SL". Vertical axis shows the chromosomes 1 to 12, which are separated by horizontal bars. Genomic regions substituted by the Kasalath genome within each CSSL are indicated in blue columns. The red horizontal bar shows the location of the locus for the tolerance to high-temperature stress determined by the QTL mapping. This figure was taken from the website of the Rice Genome Research Center (https://www.rgrc.dna.affrc.go.jp/data/NK-SL54-20030430.pdf), where detailed information of the CSSLs was available.

Supplementary Table S1. List of primers used for the SSR markers

Name	Chr.	Location		Primers
RM14327	3	1.49	F	5'-GATGCAGTAGGAACACCAAACAGC
			R	5'-ATCGAGTACCAAGTGCCTGTGC
RM14349	3	1.93	F	5'-GATCCTTAGGCATGGAATGATGG
			R	5'-CGTGTTCACTGAATATGGGAAAGC
RM14378	3	2.41	F	5'-AGTAGGTGGCTGCATTGGTTTGC
			R	5'-GTGGCTTACCTCGAGGGAGAAGG
RM14395	3	2.84	F	5'-AATATCCGCAGCCGAAACATAGC
			R	5'-ACCGACCAAACCAACACAATCG
RM14415	3	3.18	F	5'-CTCGGAAACTTCCTTTCCTTTCC
			R	5'-TCATCTCGTCAACCACGTCTCC
RM14423	3	3.25	F	5'-AGTCAGTCAGTCCAATTCAGTCACG
			R	5'-AGGACGACGACGAGTGTAACTGC
RM6849	3	3.27	F	5'-CGTCAACTGCATCACCACC
			R	5'-TCCGACTGATCATCATCGAC
RM14427	3	3.34	F	5'-CTCGCGAGCTCACATCTGTTGC
			R	5'-CCACCTGCACTGCACATACACG
RM14430	3	3.36	F	5'-AAGCAACGCAAGAACCCTTGG
			R	5'-ATGGCCGTCCAATAAACCAACC
SSR352	3	3.52	F	5'-CCCCAAATCTCGTTTTCTGC
			R	5'-GCTGCCTTTCCTTTCGTCTT
RM3392	3	3.81	F	5'-GTCCAATGATTCGTTCCCAC
			R	5'-CTTCACCGTTCACCAATTCC
R3361	3	4.31	F	5'-CGCCATCTTCCTCACTA
			R	5'-ATATTATCGGTCGCCTTTT

Names: name of SSR markers, Chr.: chromosome number,
Locaion: physical distance (Mb), Primers: forward (F) and reverse (R) primers

Supplementary Table S2. Evaluation of grain appearance quality of CSSLs

	no. of grains	no. of mitigated grains	Ratio(%)
Niponbare	41	0	0.0
SL1	36	4	11.1
SL2	40	2	5.0
SL3	34	8	23.5
SL4	45	3	6.7
SL5	35	18	51.4
SL6	40	13	32.5
SL7	48	0	0.0
SL8	46	9	19.6
SL9	41	0	0.0
SL10	35	12	34.3
SL11	36	8	22.2
SL12	40	10	25.0
SL13	44	31	70.5
SL14	40	23	57.5
SL15	34	11	32.4
SL16	46	3	6.5
SL17	39	9	23.1
SL18	43	10	23.3
SL19	46	5	10.9
SL20	43	1	2.3
SL21	48	26	54.2
SL22	38	21	55.3
SL23	48	8	16.7
SL24	45	11	24.4
SL25	40	1	2.5
SL26	38	0	0.0
SL27	35	0	0.0
SL28	38	0	0.0
SL29	38	10	26.3
SL30	35	5	14.3
SL31	42	0	0.0
SL32	36	18	50.0
SL33	35	12	34.3
SL34	40	8	20.0
SL35	36	9	25.0
SL36	34	24	70.6
SL37	44	30	68.2
SL38	38	10	26.3
SL39	40	0	0.0
SL40	42	23	54.8
SL41	43	22	51.2
SL42	40	18	45.0
SL43	38	6	15.8
SL44	36	9	25.0
SL45	38	2	5.3
SL46	38	20	52.6
SL47	45	0	0.0
SL48	37	9	24.3
SL49	32	10	31.3
SL50	37	7	18.9
SL51	35	11	31.4
SL52	36	22	61.1
SL53	33	1	3.0
SL54	39	3	7.7

map	Japonica	Indica	polymorphism	transcript	annotation
3.402	Os03g0162800	OsI_10129	ND	XM_015774439	β-ketoacyl-CoA synthase (OsCUT3)
3.408	Os03g0162900	OsI_10130	ND	AK099687	PPR protein
3.417	Os03g0163100	OsI_10131	ND	AK0655571	DUF1012 protein
3.425	Os03g0163200	OsI_10132	ND	AK108613	DUF247 protein
3.427	Os03g0163300	OsI_10132	ND	AK103418	glutathione reductase (fragment)
3.433	Os03g0163400	OsI_10134	SNP	no report	DUF1677 protein
3.437	Os03g0163500	OsI_10135	ND	AK067219/AK072441	Exocyst complex subunit Sec15-like family protein
		OsI_10137	non in japonica		
		OsI_10138	non in japonica		
		OsI_10139	non in japonica		
		OsI_10140	non in japonica		
		OsI_10141	non in japonica		
3.470	Os03g0164300	OsI_10142	ND	AK070845	hypothetical protein
3.472	Os03g0164400	OsI_10143	splicing variant	AK063366	hypothetical protein
		OsI_10144	non in japonica		
3.479	Os03g0164700	OsI_10145	ND	AL289019	Carbohydrate/purine kinase domain containing protein
3.483	Os03g0164800	OsI_10146	ND	no report	similar to SKIP interacting protein 8 (fragment)
3.490	Os03g0165000	OsI_10148	splicing variant	C7J0A2	DNA topoisomerase III-α
3.501	Os03g0165100	OsI_10149	ND	no report	
3.508	Os03g0165266	OsI_10150	ND	XM_02602823	Chromatin remodeling factor 730 (OsCHR730)/ sucrose non-fermenting 2 (Snf2) family protein
3.513	Os03g0165300	OsI_10151	ND	AK099829/AK068034 /AL105752	Bell-like homeodomain-containing transcription factor
3.522	Os03g0165375		non in indica	AJ234427	similar to chlorophyll a-b binding protein 2 (chloroplastic)
3.525	Os03g0165400	OsI_10152	ND	AK102192	β-galacosidase (IsBgal1)
3.540	Os03g0165600	OsI_10153	fragment (ND)	AK062304(fragment)	ubiquitin carbox-terminal hydrolase 2 family protein
3.522	Os03g0165800	OsI_10154	ND	AK069165	similar to Casicle-associated membrane protein 725 (VAMP725)
3.556	Os03g0165900	OsI_10155	ND	AK102116	tetratricopeptide-like helical domain containing protein
3.562	Os03g0166000	OsI_10156	ND	AK072498	Alba, DNA/RNA-binding family protein
3.567	Os03g0166100	OsI_10157	non in indica	no report	similar to Proly 4-hydroxylase
3.570	Os03g0166200	OsI_10158	fragment	no report	similar to Oxidoreductase
3.578	Os03g0166401		non in indica	no report	no data
		OsI_10159	non in japonica		
3.600	Os03g0166600	OsI_10160	ND	no report	similar to ER glycerol-phosphate acyltransferase
		OsI_10161	non in japonica		
3.617	Os03g0166800	OsI_10162	ND	AK100637	Carbon catabolite repressor 4 (OsCCR4b)
3.625	Os03g0167000		non in indica	no report	
		OsI_10163	non in japonica		
3.631	Os03g0167100	OsI_10164	ND	CT835143 (IIndica)	no data
3.631	Os03g0167200	OsI_10165	SNP/splicing variant	AK066554	IQ-motif containing protein
3.638	Os03g0167400	OsI_10166	ND	AK358357	DUF620 protein
3.647	Os03g0167500	OsI_10167	SNP	AK070468	transcript Receptor-like membrane Ring-H2 protein (OsRMR1)
3.654	Os03g0167600	OsI_10168	SNP	AK121254	Fatty acyl-ACP recudtase, regulation of pollen exine and anther cuticle development (OsDPW1/OsMS2)
3.657	Os03g0167700	OsI_10169	ND/splicing variant	AF283668	Serine/threonine protein phosphatase 2A catalytic subunit (OsPP2A)
3.665	Os03g0167800	OsI_10170	splicing variant	AK121310	Kelch-type β-propellar domain containing protein
3.675	Os03g0168000	OsI_10171	splicing variant	AK072898	Major facilitator superfamily, general substrate transporter domain containing protein
3.678	Os03g0168100	OsI_10172	SNP	AK121575	Late embryogenesis abundant prottein repeat containing proteim (OsLEA16)
3.681	Os03g0168200		non in indica	AK099530	similar to F16A14.21
3.683	Os03g0168300	OsI_10173	ND	AK069941	DUF1997 protein
3.690	Os03g0168400	OsI_10174	ND	AK287426 (partial)	Floury Endsperm 10 (OsFLO10)
3.695	Os03g0168500	OsI_10175	SNP/splicing variant	no report	hypothetical protein
3.700	Os03g0168550	OsI_10176	splicing variant	no report	similar to PPR domain protein
3.702	Os03g0168600	OsI_10177	ND	no report	Cytochrome P450 (OsCYP704B2)
3.705	Os03g0168700	OsI_10178	ND	no report	SEST domain containing protein
3.707	Os03g0168800		non in japonica	AK288126	hypothetical protein

3.711	Os03g0168900	OsI_10179	ND	AK121913	Ribosomal L32 like protein (OsGERLP)
3.714	Os03g0169100	OsI_10180	ND	AK066306/AL061772/ AK099215	Ribulose-phosphate 3-epimerase (Pentose-5-phosphate 3-epimerase) (PPE/RPE/R5P3E)
3.717	Os03g0169000	OsI_10181	ND	AK107189	Growth regulator-related protein (OsGRRP)
		OsI_10182	non in japonica		
3.724	Os03g0169300	OsI_10183	ND	AK242896	Pentatrippeptide repeat domain containing protein
3.728	Os03g0169400	OsI_10184	ND	AK068500	Ribosomal protein L18/L5 domaing containing protein
3.729	Os03g0169500	OsI_10185	splicing variant	AK287855	Cellulose synthase like protein A4 (OsCSLA4)
3.739	Os03g0169600	OsI_10186	splicing variant	AK060900/AK104523	Dof transcription factor (OsDof12)
3.740	Os03g0169700		non in indica	AK066912	antisense transcript of OsDof12
3.744	Os03g0169800	OsI_10187	ND	AK068278	HNH endonuclease domain-containing protei, white stripe leaf 9 (OsWSL9)
3.748	Os03g0169900	OsI_10188	ND	AK063222	Pathogenic type II effector avirulence factor Avr cleavage site domain containing protein (RIN4)
3.753	Os03g0170000		non in indica		non-conding RNA
3.757	Os03g0170100	OsI_10189	ND	CT835103 (Indica)	hypothetical protein
3.761	Os03g0170200		non in indica	AK067518	similar to MADS-box transcriptio factor 30
3.764	Os03g0170300	OsI_10190	splicing variant	AK067530	3-dehydroquinate synthase family protein
3.767	Os03g0170400	OsI_10191	ND	AK242042	Receotir-like cytoplasmic kinase, Xa21-mediated disease resistant to Xoo (OsRLCK102)
3.768	Os03g0170500	OsI_10192	ND	CT835093 (indica)	similar to putative eribonuclease P
3.785	Os03g0170600	OsI_10193	SNP/fragment	AK108366	similar to homeobox leucine zipper protein HOX21 (OsHOX21)
3.786	Os03g0170701		non in indica	CT837907 (Indica)	hypothetical protein
3.794	Os03g0170800	OsI_10194	ND	no report	hypothetical protein
3.798	Os03g0170900	OsI_10195	Indel in promoter	AK109355	sucrose transport 1 (OsSUT1)
3.814	Os03g0171300		non in indica	AL069569	Atypical non-DNA-binding bHLH protein (PGL1, ILI6)

Supplementary Table S3. List of genes predicted in the 3.36-3.81 Mb region of chromosome 3. Genes in the japonica and indica genomes were obtained from the nucleotide sequences of cv. Nipponbare (acc. no. AP008209) and cv. 93-11 (acc. no. CM000128), respectively. The maps and names of genes are indicated by those shown in the database. In the polymorphism column, "ND" shows no differences in the coding and putative promoter regions. "Indel in promoter" indicates an insertion/deletion in the promoter region but no differences in the coding sequence. "SNP" indicates genes with some differences in the coding region, resulting in difference in its amino acid sequences. Genes indicated with "splicing variant" are predicted to have the same nucleotide sequence but some differences in its splicing sites. "non in indica" and "non in japonica" indicate genes predicted only in japonica and indica, respectively. "fragment" is an ORF without an initiation codon. The transcript column shows the accession numbers of the Japonica cDNAs of the corresponding genes. Annotation of the predicted gene product is shown.
